# Nanoparticulated WO_3_/NiWO_4_ Using Cellulose as a Template and Its Application as an Auxiliary Co-Catalyst to Pt for Ethanol and Glycerol Electro-Oxidation

**DOI:** 10.3390/ijms25020685

**Published:** 2024-01-05

**Authors:** Munique G. Guimarães, Julio L. Macedo, José J. Linares, Grace F. Ghesti

**Affiliations:** 1 Laboratory of Bioprocesses Brewing Technology and Catalysis in Renewable Energy, Institute of Chemistry, University of Brasilia, Brasilia 70910-900, DF, Brazil; muniquegg@gmail.com (M.G.G.); julio@unb.br (J.L.M.); 2 Laboratory of Chemical Processes Development, Institute of Chemistry, University of Brasilia, Brasilia 70910-900, DF, Brazil; joselinares@unb.br

**Keywords:** nanocrystalline cellulose, microcrystalline cellulose, nickel oxide, tungsten trioxide, electrocatalysis, ethanol, glycerol

## Abstract

This work reports the use of cellulose as a template to prepare nanosized WO_3_ or NiWO_4_ and its application as a co-catalyst in the electro-oxidation of ethanol and glycerol. Microcrystalline cellulose was hydrolyzed with phosphotungstic acid (H_3_PW_12_O_40_) to prepare the nanocrystalline cellulose template. The latter was air-calcinated to remove the template and obtain nanometric WO_3_. Tungsten oxide was impregnated with Ni(NO_3_)_2_, which was subsequently air-calcinated to obtain the nanometric NiWO_4_. Elemental analysis confirmed the coexistence of nickel and tungsten, whereas thermal analysis evidenced a high thermal stability for these materials. The X-ray diffractograms displayed crystal facets of WO_3_ and, when Ni(II) was added, NiWO_4_. The transmission electron micrographs corroborated the formation of nanosized particles with average particle sizes in the range of 30 to 50 nm. Finally, to apply this material, Pt/WO_3_-C and Pt/WO_3_-NiWO_4_-C were prepared and used in ethanol and glycerol electro-oxidation in an alkaline medium, observing a promotional effect of the oxide and tungstate by reducing the onset potential and increasing the current density. These materials show great potential to produce clean electricity or green hydrogen, contributing to energetic transition.

## 1. Introduction

Biomass is gaining more relevance in the worldwide energy panorama [1]. In the transition toward a sustainable energy reality, biomass is expected to account for up to 66% of the renewable energy market. Biomass processing produces waste whose treatment and valorization are necessary to make its life cycle sustainable [2]. For example, every ton of sugarcane processed produces 280 kg of bagasse [3,4], which is primarily used to generate energy in the sugar/ethanol industry [5]. However, other alternatives are possible, including the production of second-generation ethanol [6,7] or nanocrystalline cellulose (NCC) [8]. Phosphotungstic acid (PWA) is a promising material for the hydrolysis of cellulose fibers to obtain NCC due to its low toxicity, easy handling, high Brønsted acidity, and thermal stability [9].

NCC has many applications in medicine, film preparation, sensors, water purification, packaging, polymer composites, energy storage, catalysis, and environmental remediation [10,11]. One particular application of NCC is its use as a template for preparing metal oxides with controlled size, structure, and porosity, as recently reviewed by Anžlovar and Žagar [12], such as TiO_2_ (the most extendedly prepared), ZnO, Fe_2_O_3_, CuO, Mn_3_O_4_, Nb_2_O_5_, Co_3_O_4_, and SnO_2_ and mixed oxides, such as Cu_0.5_Co_0.5_Fe_2_O_4_ or BaFe_12_O_19_/CoFe_2_O_4_. The process consists of impregnating the NCC with a metal precursor and applying a thermal treatment in an air atmosphere to obtain the metal oxide, or, even in the absence of air (pyrolysis), to prepare a metal oxide supported on carbon. These compounds can be applied in catalysis, microelectronics, environmental remediation, energy storage, ceramic fabrication, medicine, and sensors [13]. Energy and environmental applications are becoming more relevant due to the urgent necessity of changing the current energy panorama from mostly fossil to renewable sources [14].

In this sense, fuel cells and electrolyzers are receiving more attention due to the possibility of producing energy or green hydrogen from renewable sources. Although hydrogen is widely used in fuel cells, some shortcomings are still associated with its production, primarily from fossil fuels [15], safety, and storage [16]. In the case of water electrolysis, the main limitation comes from the high value of the produced hydrogen due to the requirement of high cell voltages [17]. The replacement of the oxidant, hydrogen in fuel cells, or water in electrolyzers with liquid alcohols, could partially solve the storage, transportation, and safety issues. These substitutions give rise to direct alcohol fuel cells and alcohol electroreformers, producing electricity and hydrogen from the liquid alcohol electro-oxidation. In particular, ethanol and glycerol are of interest as they come from sugarcane fermentation [18] and biodiesel synthesis [19], respectively.

Ethanol and glycerol electro-oxidation undergo sluggish kinetics. This is due to complex oxidation mechanisms involving several steps: alcohol adsorption, dehydrogenation, the formation of strongly adsorbed alcoholic species on the surface of the catalyst, the addition of oxygenated species from neighboring active sites, and C-C cleavage [20]. At the low temperatures used in the polymer electrolyte membrane fuel cells/electroreformers, platinum (Pt), or platinum-based electrocatalysts are recognized as the reference material, given their high catalytic performance [21,22,23]. Nonetheless, Pt is expensive and scarce, as well as severely deactivated from the adsorbed residues produced during the alcohol electro-oxidation. This shortcoming can be overcome by the addition of metal oxides as co-catalysts to Pt based on a double promotional effect, including (a) an electronic effect, in which the electron interaction between metal oxides and Pt alters Pt’s electronic structure, reducing the adsorption strength of the adsorbates [24], and (b) a bifunctional mechanism, where the oxyphilic oxides can donate OH_ads_ species at a lower potential, refreshing the Pt surface [22]. One metal oxide with an intense promotional effect, both in acid and an alkaline medium for alcohol electro-oxidation, is nickel oxide [22]. Furthermore, we can add an extra promotional effect using oxides with a prominent spillover effect, such as WO_3_ [25,26,27,28,29], in which WO_3_ can uptake the Pt-H_ads_ formed in the initial stages of ethanol and glycerol electro-oxidation, in addition to the supply of OH_ads_ species. Moreover, if nickel and tungsten precursors are calcinated together, nickel tungstate (NiWO_4_) (the binary metal oxide NiO/WO_3_) can be formed, a material with high stability and low corrosion that is electrocatalytically active [30,31]. Indeed, NiWO_4_ has been applied as a catalyst for hydrogen and oxygen evolution reactions [32,33,34], as well as hydrogen oxidation and oxygen reduction in an acidic medium [35] and alkaline medium [36], acting as a co-catalyst with Pt. To the authors’ knowledge, there is a sole application to the methanol electro-oxidation in an acid medium, proposed by Adzic and Marinkovic [37], with no further exploration of its potential for alcohol oxidation.

With these antecedents, this study aims to prepare multifunctional catalysts based on nanometric metal oxides, WO_3_ (labeled hitherto TO) and NiWO_4_ (labeled hitherto NiT), using nanocrystalline cellulose (NCC) as a template. NCC was prepared from the acid hydrolysis of microcrystalline cellulose (MCC) using phosphotungstic acid (PWA, H_3_PW_12_O_40_). The prepared materials were characterized by CHN analysis, Energy Dispersive Spectroscopy (EDS), X-ray diffraction (XRD), and Transmission Electron Microscopy (TEM). In addition, Pt nanoparticles were deposited on a mixture of metal oxide and carbon support to improve electrical conductivity. The obtained catalysts were tested for ethanol and glycerol electro-oxidation in an alkaline medium to evaluate their performance and potential applications in these relevant electrochemical reactions.

## 2. Results

### 2.1. Nanocrystalline Cellulose

**Figure 1:**
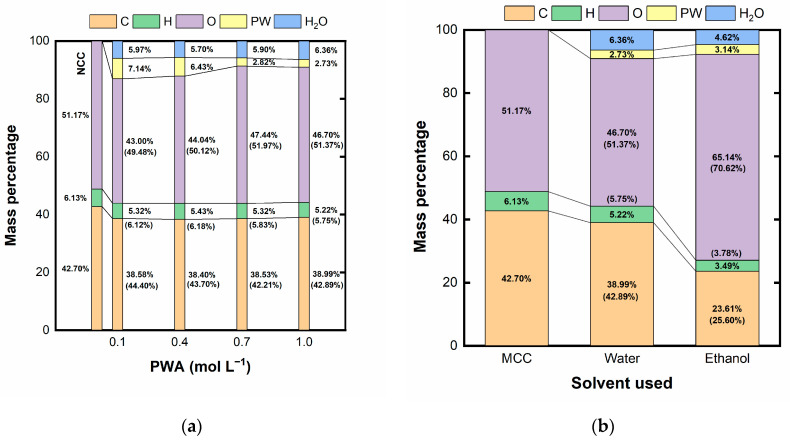
Mass percentage of the different elements as a function of (**a**) concentration of PWA in water, and (**b**) the solvent used in the hydrolysis for 1 mol L^−1^ of PWA (PW is the fraction of phosphorous and tungsten from the PWA; values in parenthesis represents the relative percentages of C, H, and O to compare with the original MCC).

Table S1 (Supplementary Materials) summarizes the primary immediate analysis of the raw matter used in this study, MCC. The most relevant result is the absence of ash material. This is important considering that the subsequent NCC used as a template is expected not to leave any residue in the prepared electrocatalyst. Figure 1 collects the corresponding results of the final composition of the NCC after acid hydrolysis with PWA.

As observed in Figure 1a, the aqueous acid hydrolysis inserted phosphorous and tungsten (identified as PW) in the NCC due to the PWA used in the procedure. In parallel, the amount of PW that remained in the NCC structure reduced as the PWA hydrolysis concentration increased, without significant variations in the C, H, and O percentages compared to MCC. On the other hand, the results displayed in Figure 1b showed a significant drop in C percentage accompanied by an increase in O percentage when ethanol was used as solvent with a PWA concentration of 1 mol L^−1^. Moreover, there was an increase in PW percentage, augmenting from 2.73 to 3.14% when water was replaced by ethanol.

Figure S1 displays MCC and NCC (hydrolysis in water using 1 mol L^−1^ of PWA) images obtained from TEM measurements. The hydrolysis procedure resulted in a significant reduction in the particle size. The use of lower PWA concentrations led to larger NCC particles. For this reason, the NCC prepared with a 1 mol L^−1^ aqueous solution of PWA was chosen for subsequent characterizations.

The thermal stability of MCC and NCC was compared in Figure S2, exhibiting similar TG and DTG curves. However, there was a slight decrease in the onset temperature of the polysaccharide degradation for the NCC material (327.5 °C) compared to MCC (351.0 °C). This drop in thermal stability can be associated with a decrease in crystallinity after acid hydrolysis. Also, the presence of P and W on the NCC surface could catalyze an earlier decomposition.

Figure S3 shows the XRD patterns of the MCC and NCC (hydrolysis in water using 1 mol L^−1^ of PWA) materials. As observed, both samples presented similar diffraction peaks, indicative of the preservation of the basic crystalline structure after acid hydrolysis. No signals associated with PWA were visible, likely due to a high dispersion of the acid on the NCC surface.

### 2.2. Nanosized NiT/TO

**Figure 2:**
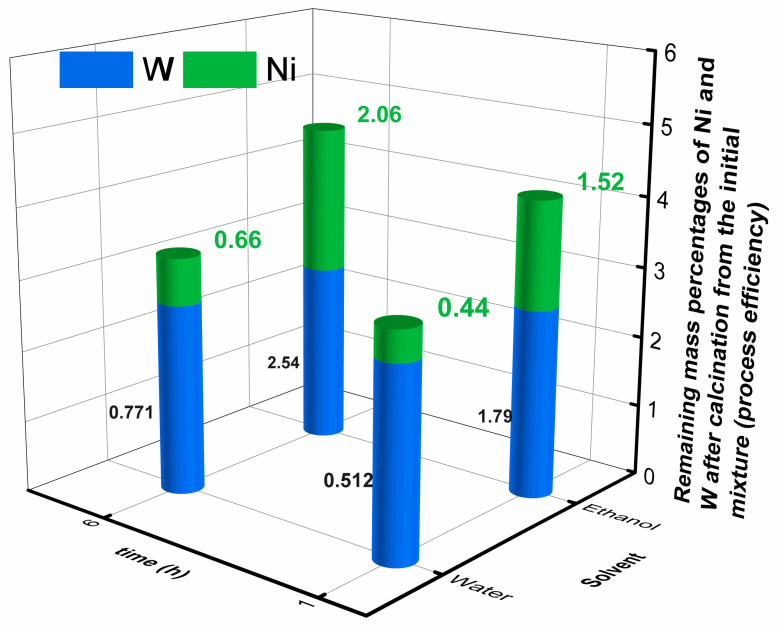
Ni and W remain after the impregnation and calcination steps. Numbers in the upper part of the bars represent the percentage of Ni deposited from the initial sample. In contrast, the numbers in the center of the blue bar represent the Ni/W atomic ratio.

The synthesis of binary metal oxides was studied from the Ni(NO_3_)_2_-impregnated NCC (hydrolysis in water using 1 mol L^−1^ of PWA) samples prepared with different impregnation times (1 and 6 h) and solvents (ethanol and water). Calcination was performed at 600 °C for 8 h to guarantee the NCC template decomposition (see Figure S2). Figure 2 shows the metal weight percentages of Ni and W remaining in the calcinated samples, evidencing the efficiency of the nickel anchorage on the NCC surface. In addition, no phosphorous was detected in the calcinated samples, indicating that it probably evolved as P_2_O_5_ and lixiviated during the washing process as phosphoric acid.

As observed, the highest amount of Ni was obtained when ethanol was used as a solvent. Given that this work aimed to obtain a binary metal oxide with the highest Ni content and an equilibrated Ni/W atomic ratio, the sample prepared in ethanol (2.06% of Ni) with 1 h of impregnation time (1.79 Ni/W ratio) was the most suitable condition.

**Figure 3:**
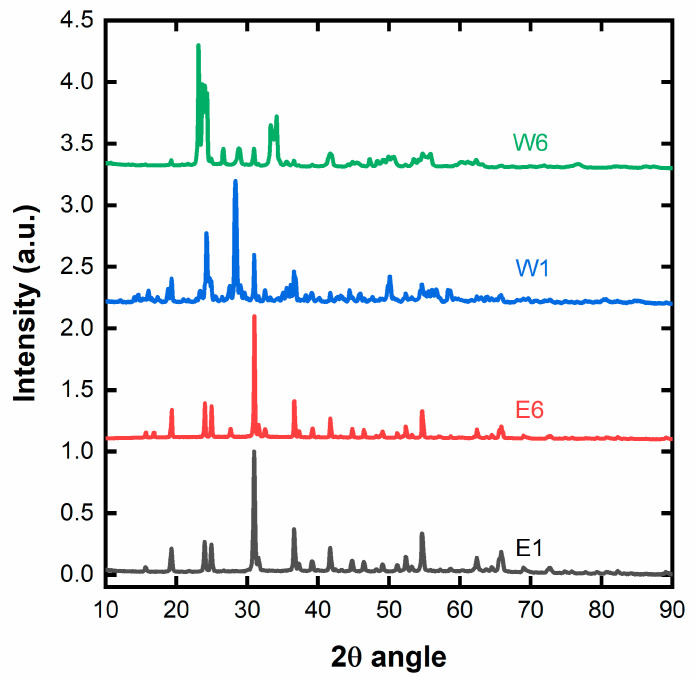
XRD pattern of the different mixed oxide samples as a function of the solvent and time.

Figure 3 displays the XRD diffraction patterns of the prepared mixed oxides after calcination, where letters E and W indicate the use of ethanol or water as a solvent, respectively, and numbers 1 and 6 indicate impregnation time. Figure S4 shows the characteristic peaks and planes of NiO (ICSD n. 112324), TO (monoclinic, ICSD n. 17003), and NiT (ICSD n. 16685). As can be observed, all the materials were formed as a combination of TO and NiT, with no segregated NiO phases. The proportion and intensity of each phase depend on the amount of each metal present in the final calcinated product. Also, the solvent and time used to anchor the Ni precursor on the NCC impact the detected phases. The XRD of E1 and E6 samples showed the monoclinic crystal structure of NiT (see Figure S4), with the most intense peaks at 19.3, 24.0, 24.9, 31.0, 36.6, 41.8, 52.4, and 54.7° corresponding to the (100), (011), (110), (111), (002), (121), (130), and (202) planes of NiT [38,39]. In the case of the W1 material, the most intense peak, at 28,3°, is attributed to the (200) plane of the hexagonal crystal structure of TO [40]. Finally, the W6 material presents the highest peak at 23.1 in a sequence of three consecutive intense peaks. Such a pattern fits well with the monoclinic TO [41]. In summary, all samples display peaks attributable to TO in a combination of orthorhombic, monoclinic, and hexagonal phases, along with NiT. Table 1 collects the corresponding average crystallite size after applying Scherrer’s equation to the three most intense peaks of each sample with the corresponding error bars [42].

**Figure 4:**
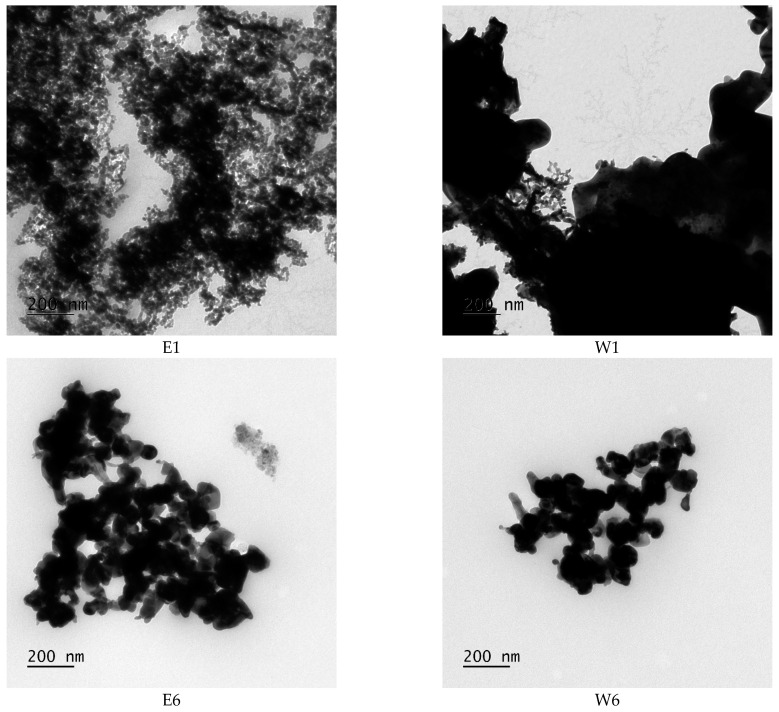
TEM images of the E1, E6, W1, and W6 materials.

Figure 4 shows the TEM images of the calcinated materials obtained with different impregnation conditions (E1, E6, W1, and W6). As observed, in accordance with the XRD results, the sample prepared with ethanol and impregnated for 1 h presented the smallest and most homogeneous particle sizes and distribution compared to the other materials. The average crystallite size (Table 1) and TEM micrographs confirmed that the conditions used to prepare the E1 sample led to the smallest crystalline and particle sizes and the most homogeneous particle distribution.

### 2.3. Pt/TO-C and Pt/NiT-C Electrocatalysts

**Figure 5:**
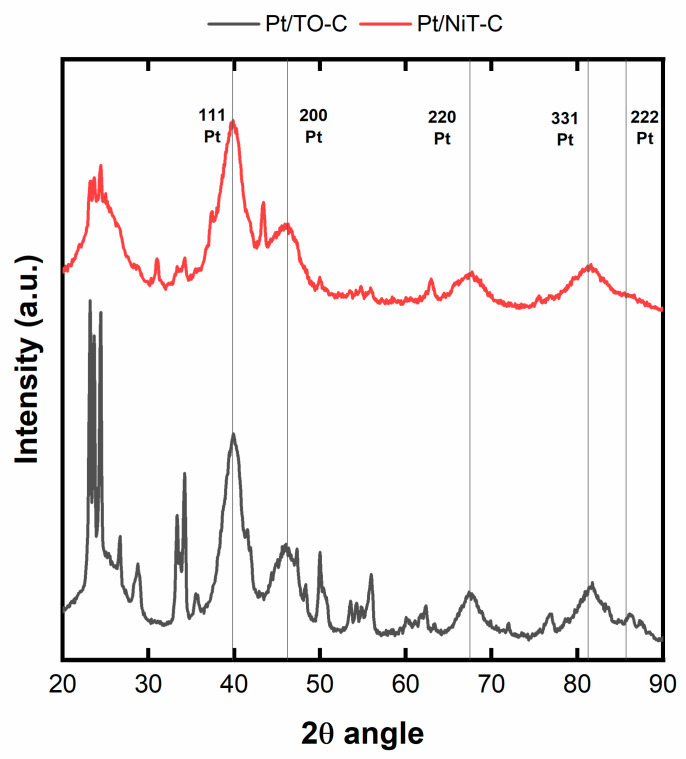
XRD pattern of the Pt-based electrocatalysts prepared on the TO-C and NiT-C supports (lines represent the main Pt fcc diffraction peaks).

Figure 5 displays the XRD pattern of the prepared Pt/TO-C and Pt/NiT-C (combined supports of TO and C, and NiT and C, respectively) electrocatalysts. As can be observed, the typical peaks attributed to the Pt fcc (111), (200), (200), (311), and (222) were present at 39.8, 46.2, 67.5, 81.3, and 85.7°, respectively. The presence of these peaks confirmed the Pt deposition on the combined support of carbon and metal oxide. The average crystallite sizes are 3.2 and 2.5 nm, estimated using Scherrer’s equation. In the Pt/TO-C, high peaks at 2θ angles of 25 and 34° also stand out, as well as the sequence of peaks between 50 and 60°, attributed to TO (Figure S4). In the case of the Pt/NiT-C, peaks associated with the (111), (002), (121), and (202) NiT facets appear at approximately 2θ angles 31, 37, 43, and 54°, respectively, along with the peaks of TO (see also Figure S4), which is indicative of the presence of the two phases in the Pt/NiT-C material. It is relevant to note that the diffraction peaks of the TO/NiT in the Pt/NiT-C material are less intense than TO in the Pt/TO-C electrocatalyst.

**Figure 6:**
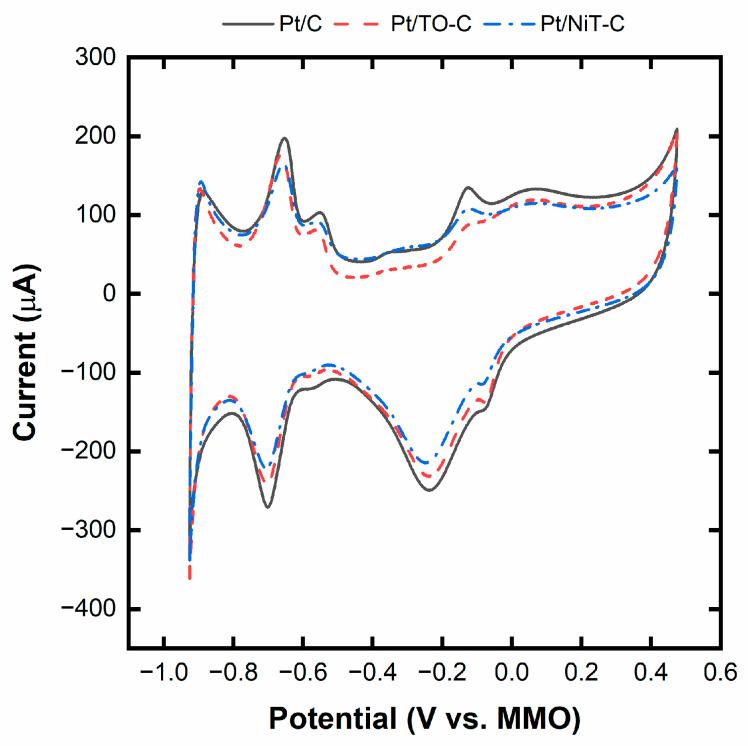
Blank voltammograms of the prepared electrocatalysts in 1 mol L^−1^ KOH.

Figure 6 displays the blank voltammograms of the prepared materials compared to the reference Pt/C. The peaks associated with the hydrogen adsorption/desorption appear for all the materials, as well as the formation of the Pt oxide (including the formation of the Pt-OH_ads_ prelayer at approx. −0.15 V vs. MMO (Hg/HgO/KOH, mercury/mercury oxide reference electrode)), and their corresponding reduction peaks in the cathodic scan.

**Figure 7:**
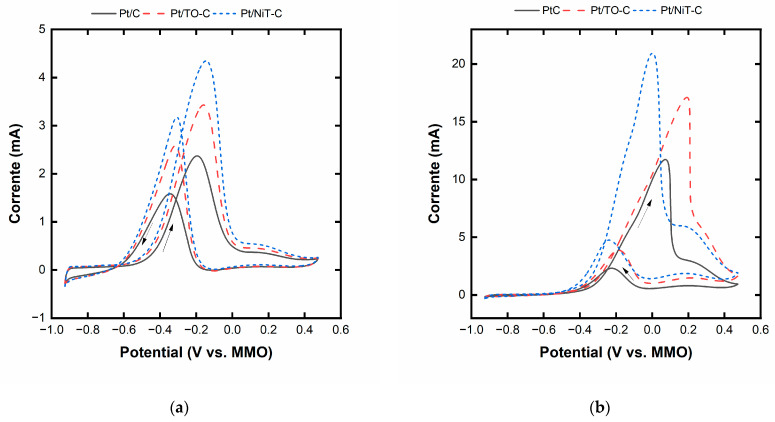
(**a**) Ethanol and (**b**) glycerol electro-oxidation curves of the different electrocatalysts in 1 mol L^−1^ alcohol and 1 mol L^−1^ KOH (arrows indicate the direction of the voltammetry).

Figure 7 displays the ethanol and glycerol electro-oxidation curves in the acid and alkaline medium for the different materials. The addition of TO, and especially NiT-TO, significantly increases the ethanol and glycerol electro-oxidation activity. To have a better insight, Table 2 collects the values of the onset potential and the maximum current for each catalyst. As can be seen, there is a decrease in the onset potential and a more evident increase in the maximum current when the tungsten oxide and nickel tungsten are added. Figure S5 shows the corresponding chronoamperometric curves for the different materials with ethanol and glycerol. As in the case of voltammograms, adding TO and NiT-TO to the catalyst formulation leads to higher currents and a lower current decay.

## 3. Discussion

The results displayed in Figure 1 evidence that the increase in the PWA concentration reduces the amount of phosphorus and tungsten that remain in the NCC structure. One possible explanation lies in the observation of Zhao et al. [43], where the acid concentration affects the macrostructure of the resulting hydrolysis products. The authors observed that an increase in the acid concentration increased cellulose hydrolysis, exposing more nanofibers due to a more aggressive treatment. Nevertheless, the increase in the acid concentration also tends to agglomerate the larger fibers of the MCC, which may reduce the contact between the MCC and the PTA, decreasing the PW percentage of the hydrolyzed samples. On the other hand, the exposed MCC surface (although smaller) undergoes more severe hydrolysis, resulting in NCCs of smaller sizes, as corroborated by the TEM images and the slight decrease in thermal stability. In the case of using ethanol as a solvent, there are more noticeable changes in the MCC to NCC transition. In particular, the O content drastically increases compared to water as a solvent. Ethanol might reduce the aggregation of the MCC fibers, as it may be able to better interact with these fibers given its lower dipolar moment, exposing more surface to the PWA attack. PWA is known to be a strong oxidizing agent [44], increasing the O percentage. For this reason, we selected aqueous PWA hydrolysis with an acid concentration of 1 mol L^−1^.

Figure 2 shows evidence that the solvent and impregnation time strongly influence the subsequent impregnation process for preparing the binary oxide NiT. The most favorable condition for maximizing the Ni percentage balanced with W is achieved after using ethanol as the impregnation solvent for 1 h. It then seems that ethanol promotes the interaction of the NCC template and the nickel precursor due to its smaller polarity, rendering a more Ni-charged material. On the other hand, an excessive impregnation time unbalances the Ni/W proportion, leading to a W-overcharged material.

The XRD patterns also confirm that more NiT is formed when ethanol is used as the solvent, as the NiT diffraction peaks emerge as the highest peak in the E1 and E6 samples, which is not the case in the W1 and W6 samples, where the peaks attributed to TO are higher than the NiT ones. Moreover, the E1 NiT sample presents the smallest average crystallite sizes, which is desirable for application in electrocatalysis as it exposes the highest surface area. For these reasons, the calcinated NiT E1 material is selected for preparing the potential Pt electrocatalyst (Pt/NiT-C) to be applied in the glycerol and ethanol electro-oxidation, in addition to Pt/TO-C. Finally, it is important to note that despite the excess of nickel in the ethanol-based samples, diffraction peaks associated with NiO are not visible. Thus, NiO could be present in the form of an amorphous oxide. The formation of this multiphase material may be of interest based on the promotional properties of TO, NiT, and NiO, which may be further boosted by a synergistic effect.

To provide electronic conductivity to the TO and NiT materials, they were mixed with carbon, and, in the sequence, Pt was deposited onto this hybrid support. The XRD patterns confirm the successful deposition of Pt in the form of small nanocrystals. These values are close to commercial 20 wt.% Pt/C (3.2 nm, Novocell, São Paulo, Brazil, https://www.novocell.ind.br/pt/produtos/componentes/material-catalitico, accessed 18 December 2023). The low particle sizes achieved are typical of the formic acid reduction method used in this work [45]. It is also important to note the presence of diffraction peaks associated with TO in the Pt/TO-C material and NiT (along with TO) in the NiT-C correspond to the crystalline arrangements of these species. Moreover, the intensity of the TO/NiT peaks in the Pt/NiT-C material is smaller than TO in the Pt/TO-C, which could be an indication of the formation of smaller nanocrystals in the NiT-C material compared to TO-C. Also, the formation of nanometric TO and NiT can help form small Pt nanocrystals.

Figure 5 displays the blank voltammograms of the prepared materials compared to the commercial Pt/C. The three materials present the same shape, with the typical peaks ascribed to the hydrogen adsorption/desorption region and the PtO formation and reduction. No apparent voltammetric signal can be assigned to TO and NiT, which is interesting from the point of view of the stability of these auxiliary species.

The ethanol and glycerol electro-oxidation curves reveal the promotional effect that TO and, especially NiT, exert on Pt. A decrease in the onset potential can be observed and is primarily attributable to the bifunctional mechanism coming from the presence of TO in the Pt/TO-C material and the mixed oxide present in the Pt/NiT-C. More noticeable is the increase in the maximum current densities. The smaller crystal size of Pt/NiT-C compared with Pt/C and Pt-TO/C may partially explain the enhanced performance of that material. Nonetheless, the combined bifunctional effect, which assists in removing more efficiently the alcoholic carbonaceous residues that adsorb onto the Pt surface, and the possible electronic effect coming from the Pt/TO and Pt/NiT interactions, explain the higher activity of Pt/TO-C (similar crystal size than Pt/C) and, especially, Pt/NiT-C [46]. Figure S6 schematically depicts the promotional effects of TO and NiT on Pt for a better illustration of these mechanisms. This favorable tendency is maintained in the chronoamperometric measurements, where the Pt/NiT-C outperforms Pt/TO-C > Pt/C, in addition to the slower current decay. Therefore, the addition of TO and, for the first time, the ethanol and glycerol electro-oxidation in an alkaline medium, NiT in the nanometric form Pt has demonstrated to possess a promotional effect, postulating Pt/NiT-C for its application in direct ethanol and direct glycerol alkaline fuel cells/electroreformers. Furthermore, potential applications of these materials could be widened to the CO_2_ electrochemical reduction coupled to glycerol or ethanol oxidation instead of water oxidation. With this, it is possible to reduce the system’s energy consumption [47,48]. Furthermore, TO and NiT could have a high potential for application in water oxidation (oxygen evolution reaction), given that these materials are prone to having oxygen vacancies capable of promoting this reaction. In this sense, promising results have been demonstrated in the literature, with tungsten oxide nanowires [49] and nanofibers [50] acting as co-catalysts with Ir/IrO_x_.

## 4. Materials and Methods

Sulfuric acid (95–97 wt.%), ethanol (99.8 wt.%), microcrystalline cellulose (powder), phosphotungstic acid (H_3_PW_12_O_40_∙xH_2_O, >99.9 wt.%), hexachloroplatinic acid (H_2_PtCl_6_∙xH_2_O, 38 wt.% Pt), and nickel(II) nitrate hexahydrate (99 wt.%) were purchased from Sigma-Aldrich (Barueri, São Paulo, Brazil). Formic acid (85 wt.%), 2-propanol (99.5 wt.%), and KOH (85 wt.%) were acquired from Dinâmica (Indaiatuba, São Paulo, Brazil). Vulcan XC-72 carbon black was obtained from Cabot Corp. (Tokyo, Japan). Glass microfiber filter paper GF/F 125 mm was purchased from Hexis Científica (Jundai, São Paulo, Brazil).

NCC was prepared by mixing in a test tube 0.15 g of MCC in 0.625 mL of ultrapure water (or ethanol) and the required amount of PWA to achieve the desired acid concentration. The reactional mixture was heated for 1 h at 80 °C. Next, the product was extracted from the tube, filtered, and washed thoroughly until neutralization of the supernatant. The produced NCC from 1 mol L^−1^ PWA was subsequently impregnated with 10 mL of a 0.2 mol L^−1^ Ni(NO_3_)_2_ ethanol or water solution and heated at 80 °C for 1 or 6 h. Afterward, the impregnated sample was calcinated at 600 °C in an air atmosphere for 6 h to render the TO or NiT material.

The Pt/TO-C and Pt/NiT-C catalysts were prepared using the formic acid reduction method. In a typical procedure, 80 mg of the support, formed by combining 30 mg of TO or NiT and 50 mg of Vulcan XC-72, were dispersed in a 0.1 mol L^−1^ formic acid solution. The mixture was heated up to 80 °C, in which the necessary volume of the Pt precursor solution (50 g L^−1^ of H_2_PtCl_6_·6H_2_O) was dropwise added to the solution. After completing the addition of the Pt precursor, the temperature was maintained for an extra hour, leaving the solution to cool down and settle for 12 h. The catalyst powder was then filtered (cellulose acetate membrane, 0.22 µm, 47 mm diameter) and thoroughly washed with boiling water. Finally, the catalysts were dried in an oven at 90 °C for 1 h.

The prepared materials were characterized by X-ray diffraction (XRD) in a Bruker D8 Focus Diffractometer (Bruker, Billerica, MA, USA) using CuK_α_ radiation (wavelength 0.15418 nm) and applying a source voltage of 40 kV and a current of 30 mA. The applied 2θ angles varied from 10 to 90° at a rate of 1° min^−1^, with a step of 0.05°. Scherrer’s equation (Equation (1)) was applied to estimate the average crystal sizes (*D*), where *λ* is the wavelength of the X-ray source, *β*_1/2_ is the width of the peak at half height, and *θ* is the angle of the peak position.

(1) $$D=\frac{0.9\lambda }{\beta_{1/2}\cos\theta }$$

Elemental analyses were performed according to the Brazilian Association of Technical Standards [51]. These analyses provided the mass percentages of the main elements in the different materials. Carbon (C), nitrogen (N), and hydrogen (H) were determined with a Perkin Elmer 2400 Series II CHN Elemental Analysis. The other components were determined using a Shimadzu X-ray Fluorescence (XRF/EDX) spectrometer (model EDX 720, Kyoto, Japan) with a rhodium tube as an X-ray source. Oxygen (O) was calculated from the results obtained from CHN and XRF/EDX.

Thermogravimetry (TG) and derivative thermogravimetry (DTG) were obtained on a TA Instruments SDT Q600 (New Castle, DE, USA) from room temperature to 1000 °C (20 °C min^−1^), using synthetic air (80 ± 0.5 of N_2_ and 20 ± 0.5 of O_2_, >99.999%) as the purge gas (flow rate of 60 mL min^−1^). The analysis was performed on alumina crucibles, and the external standard employed for calibration was sapphire.

TEM images were obtained in a JEOL 1011 microscope (Tokyo, Japan) at different magnifications in a high vacuum. For sample preparation, a mass of 1 mg of samples was dispersed in 50 mL of ethanol, depositing 1 µL of the dispersion onto the copper mesh. The samples were dried for 24 h before the analysis was executed.

Electrochemical characterization was carried out by cyclic voltammetry using a three-electrode glass cell in 1 mol L^−1^ KOH. The working electrode, a 5 mm reticulated vitreous carbon electrode, was prepared by dispersion of 4 mg of catalysts in 1 mL of 2-propanol and 10 µL of a Nafion^®^ emulsion (5 wt.% in a mixture of aliphatic alcohols). A volume of 10 µL was deposited onto the working electrode and was left to dry. The counter-electrode was a platinized gauze, and Hg/HgO/KOH (1 mol L^−1^) was used as the reference electrode. The system was thoroughly purged with N_2_ before the electrochemical measurements. The blank voltammograms were executed by cycling the potential between −0.926 and 0.424 V vs. MMO until a stable voltammetry in 1 mol L^−1^ KOH was obtained.

The alcohol electrochemical activity was carried out similarly to the blank voltammetry in 1 mol L^−1^ KOH and alcohol. In this case, the voltammetry limits were −0.8 V to 0.3 V vs. MMO, repeating the cycles until similar profiles were obtained. Finally, the chronoamperometric curves, recorded after completing the cyclic voltammetry, were carried out for 30 min at −0.25 V vs. MMO. No normalization has been applied to the electrochemical measurement, as all the prepared working electrodes possessed the same Pt mass loading.

## 5. Conclusions

NCC has been demonstrated to be a suitable template for preparing nanosized TO and NiT. NCC can be readily prepared from acid hydrolysis with PWA of MCC, with an optimum acid concentration of 1 mol L^−1^ to guarantee the formation of nanosized cellulose in an aqueous medium. This NCC acts as an effective template to prepare NiT nanoparticles after preferential impregnation with ethanol for 1 h (to obtain the highest Ni deposition and the most equilibrated Ni/W ratio), rendering a mixed material with WO_3_, NiWO_4,_ and, likely, NiO. These TOs and NiTs, when applied as co-catalysts to Pt, have evidenced a promotional effect on the ethanol and glycerol electro-oxidation in an alkaline medium, enhancing the onset potential and, mainly, the current density. In this way, the oxide or binary oxides, prepared with the aid of NCC as a template, can be postulated as effective auxiliary materials for platinum with potential application for alkaline direct ethanol and direct glycerol alkaline fuel cells/electroreformers to produce clean electricity or green hydrogen.

## Figures and Tables

**Table 1:** Average crystallite size of the different prepared oxide materials.

Sample	TO Phase (nm)	NiT Phase (nm)
E1	23.5 ± 2.3	24.8 ± 1.8
E6	43.8 ± 4.3	45.3 ± 2.6
W1	30.9 ± 2.8	31.3 ± 2.4
W6	41.1 ± 3.8	38.6 ± 2.9

**Table 2:** Onset potential and maximum current for each catalyst.

Catalyst	Onset Potential for Ethanol/Glycerol (V vs. MMO)	Maximum Current for Ethanol/Glycerol (mA)
Pt/C	−0.471/−0.327	2.3/11.7
Pt/TO-C	−0.500/−0.320	3.3/17.2
Pt/NiT-TO-C	−0.508/−0.301	4.4/20.8

## Data Availability

The data presented in this study are available upon request from the corresponding author. The data are not publicly available due to privacy.

## Supplementary Materials

The following supporting information can be downloaded at https://www.mdpi.com/article/10.3390/ijms25020685/s1.
