# Supplementary material for: Nanoparticulated WO3/NiWO4 Using Cellulose as a Template and Its Application as an Auxiliary Co-Catalyst to Pt for Ethanol and Glycerol Electro-Oxidation
Source: Int J Mol Sci. 2024 Jan 5;25(2):685. doi: 10.3390/ijms25020685 (PMC10815037; doi:10.3390/ijms25020685)
Supplement: Supplementary file 1 [file ijms-25-00685-s001.zip › ijms-2750882-supplementary.pdf]

Supplementary Materials

Nanoparticulated WO_3/NiO using microcrystalline cellulose as a template and its application as auxiliary oxides to Pt for ethanol and glycerol electro-oxidation to produce green hydrogen

Munique G. Guimarães,[1] Julio L. Macedo,[1] José J. Linares[2] and Grace F. Ghesti[1,*]

[1]Laboratory of Bioprocesses Brewing Technology and Catalysis in Renewable Energy, Institute of Chemistry, University of Brasilia, Brasilia-DF, 70910-900, Brazil. [2]Laboratory of Chemical Processes Development, Institute of Chemistry, University of Brasilia, Brasilia-DF, 70910-900, Brazil. *Correspondence: grace@unb.br.

TABLES

Table S1 reports the main thermal parameters of the microcrystalline cellulose (MCC) used as raw material for preparing the nanocrystalline cellulose (NCC). As can be observed, the final ash content of the material was null, which is crucial for its application as a template and subsequent calcination.

Table S1. Thermal parameters of the MCC.

Parameter	(%)
Moisture content (%)	4.69
Volatiles (%)	94.11
Ash content (%)	0.00
Fixed carbon content (%)	5.90

FIGURES

Figure S1 displays TEM micrographs of MCC and NCC (hydrolysis in water using 1 mol L^{-1} of PWA) materials.

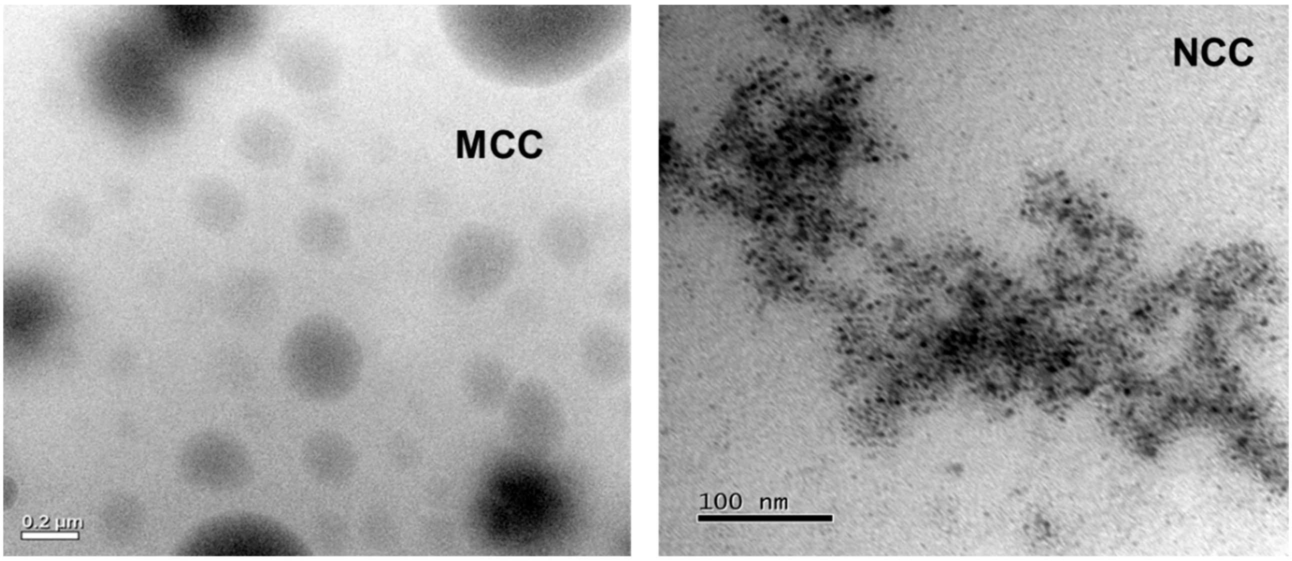

Figure S1. TEM images of MCC and NCC materials.

Figure S2 presents the TG and DTG curves of MCC and NCC (hydrolysis in water using 1 mol L^{-1} of PWA) materials. Both materials showed similar profiles, with the initial decrease in weight (up to 150 °C) associated with the

moisture content. Afterward, there was an abrupt weight decrease, beginning earlier for the NCC than for the MCC, and achieving its maximum value at 327.5 and 351.0 °C, respectively. This decrease is related to the cellulose structure's degradation, including ring scission (beginning from the β-1,4 glycosidic bonds) and decarboxylation [1,2].

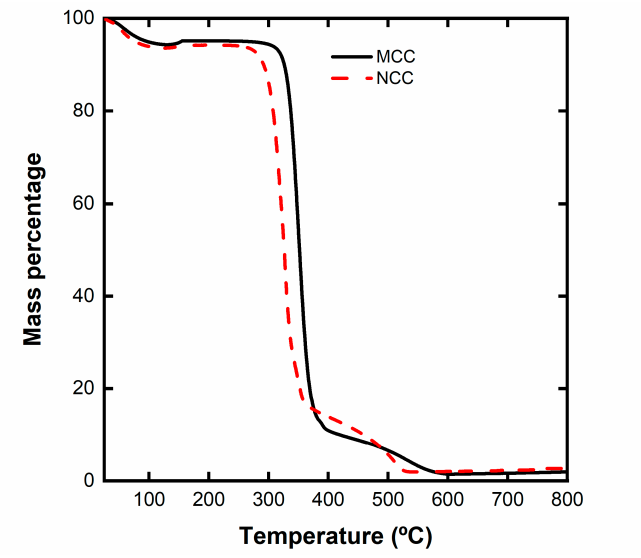

(**a**)

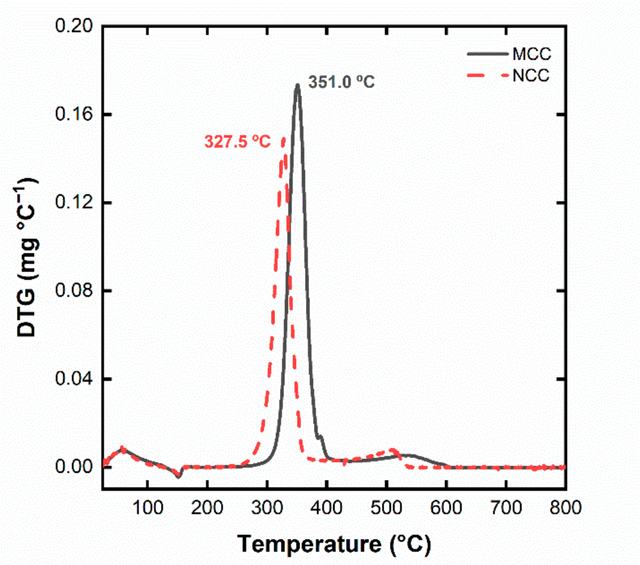

(**b**)

Figure S2. TG (a) and DTG (b) curves of MCC and NCC materials.

Figure S3 shows the XRD patterns of MCC and NCC (hydrolysis in water using 1 mol L^{-1} of PWA) materials.

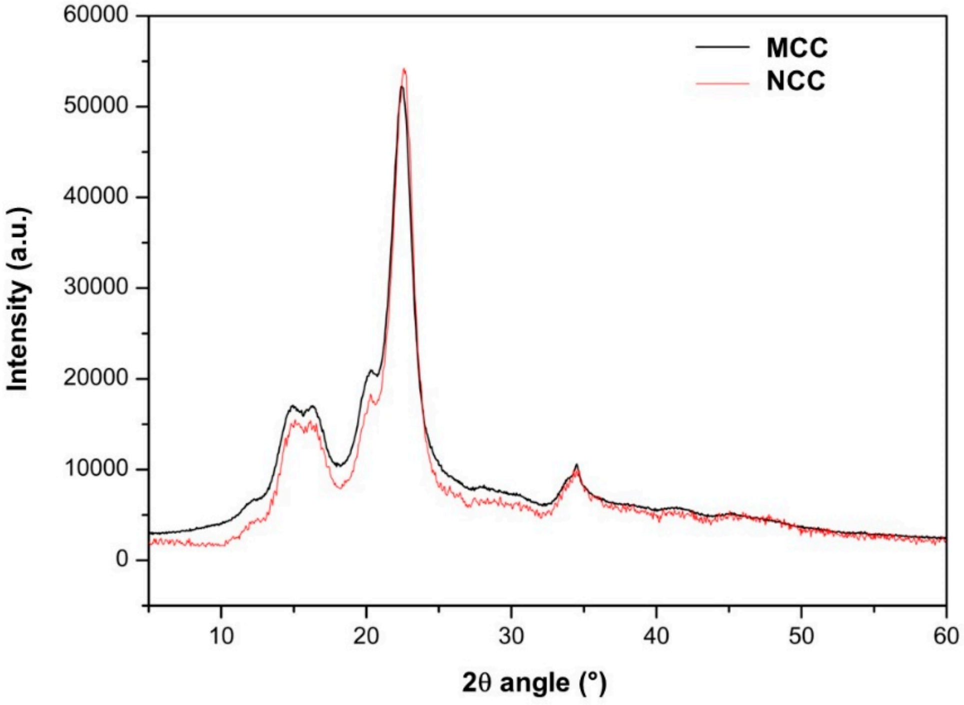

Figure S3. XRD patterns of MCC and NCC materials.

Figure S4 shows the characteristic peaks and planes of NiT (ICSD nº 16685), TO (monoclinic, ICSD nº 17003) and NiO (ICSD nº 112324).

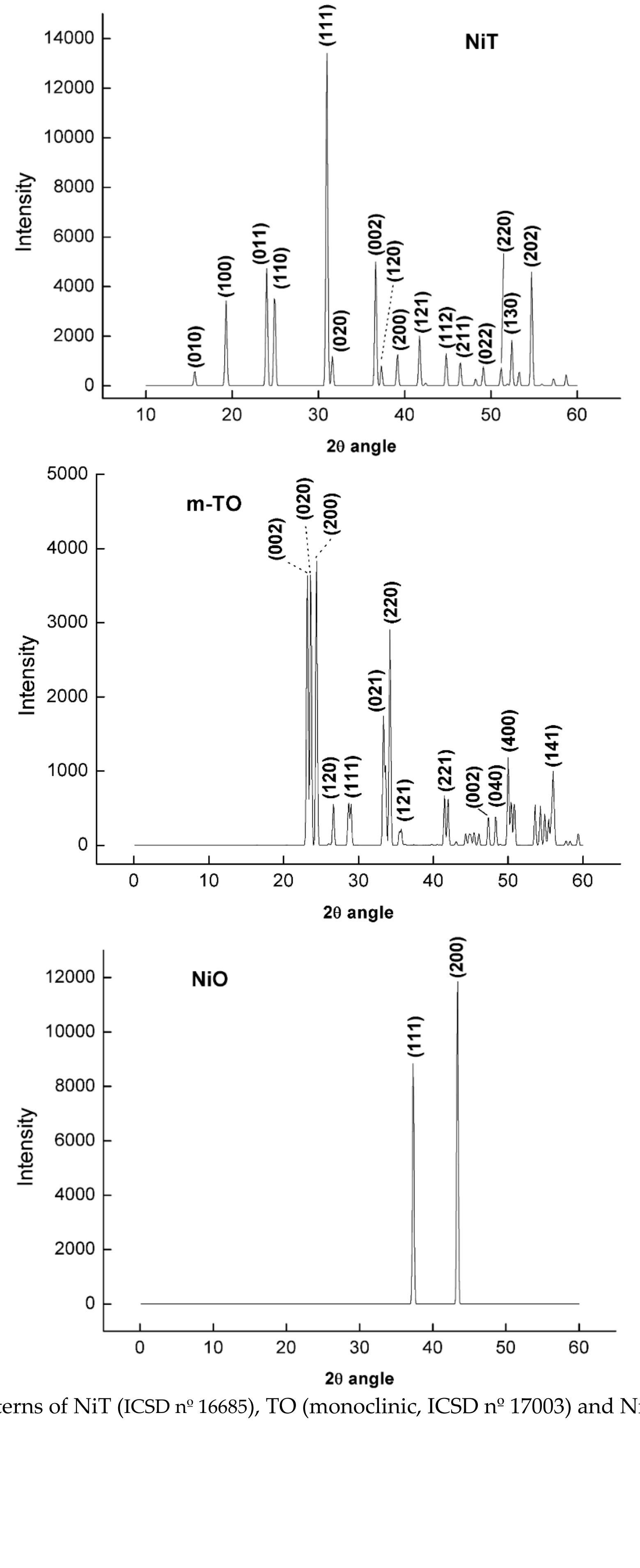

Figure S4. XRD patterns of NiT (ICSD nº 16685), TO (monoclinic, ICSD nº 17003) and NiO (ICSD nº 112324).

Figure S5 displays the chronoamperograms of the different materials for 30 min at a fixed potential of −0.25 V vs. MMO. As can be seen, the prepared materials with TO and, especially, with NiT-TO display higher current densities as a result of the positive effect of these species in terms of alleviating the accumulation of adsorbed carbonaceous residues during the ethanol and glycerol electro-oxidation.

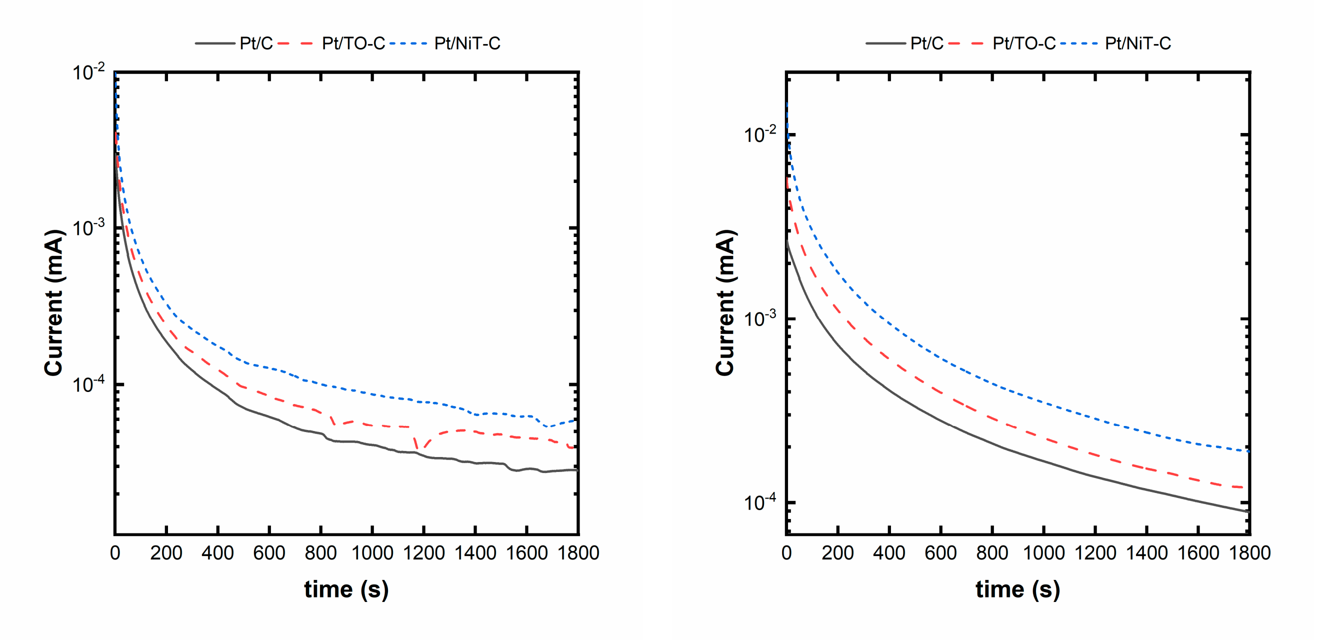

(**a**) (**b**)

Figure S5. Chronoamperometry profiles of the different materials for in 1 mol L^{-1} alcohol and in 1 mol L^{-1} KOH (a) ethanol electro-oxidation (b) glycerol electro-oxidation at a potential of −0.25 V vs. MMO.

Figure S6 schematically illustrates the promotional effects of TO and NiT according to the bifunctional and electronic effects. These mechanisms are responsible for the enhancement of the electrochemical performance of the Pt/TO-C and Pt/NiT-C electrocatalysts.

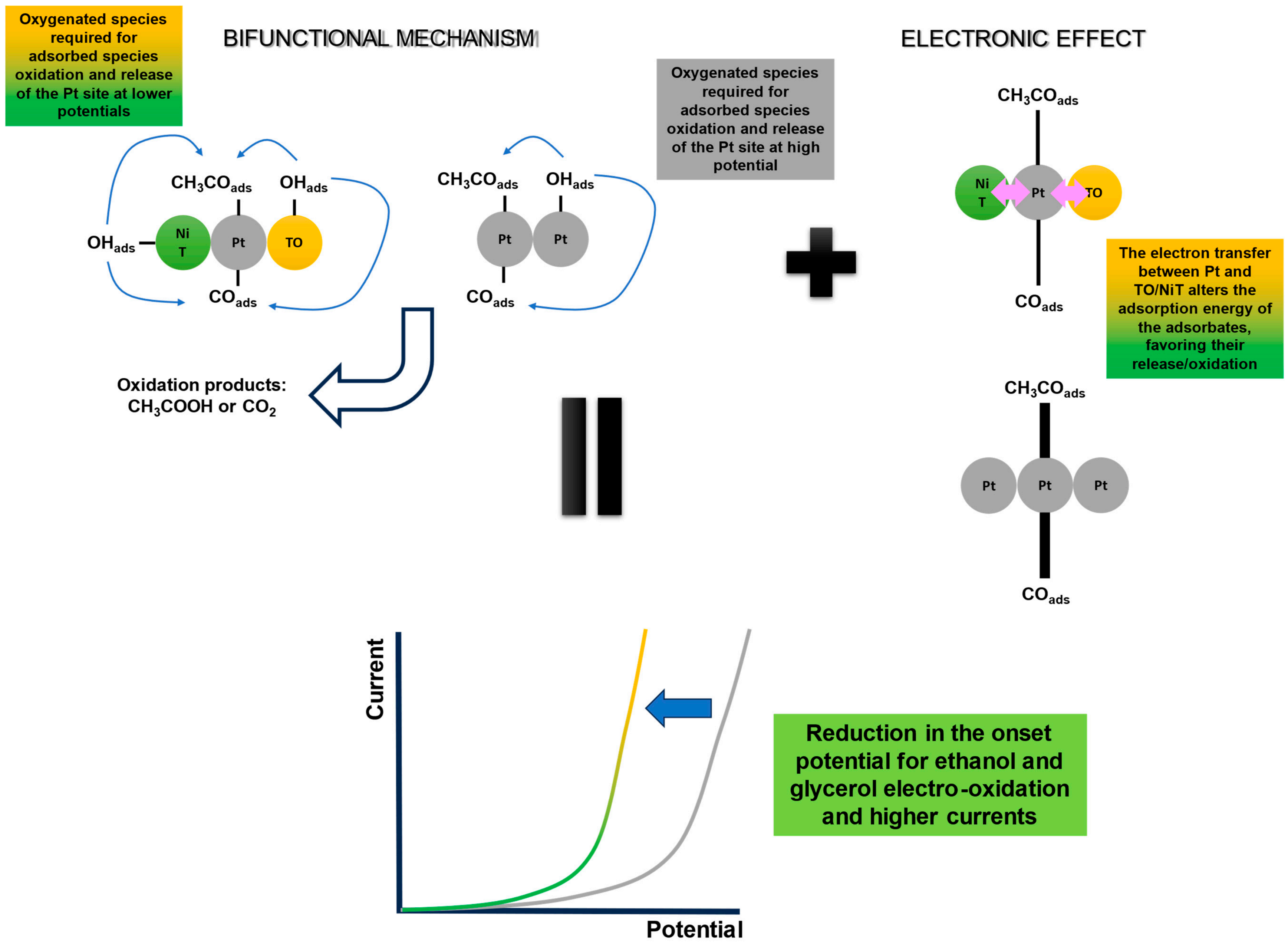

Figure S6. Scheme of the promotional effects (bifunctional mechanism and electronic effects) exerted by TO and NiT on Pt responsible for the improvement in the electrochemical performance.
